# Diagnostic utility of cardiac MRI in clinical evaluation of cardiac masses with histopathological correlation

**DOI:** 10.1186/1532-429X-14-S1-P298

**Published:** 2012-02-01

**Authors:** Rima D Patel, Ruth P Lim, Leon Axel, Monvadi B Srichai

**Affiliations:** 1 Medicine, NYU School of Medicine, New York, NY, USA

## Summary

To determine the diagnostic value of cardiac magnetic resonance imaging (CMR) in the characterization of cardiac masses in a tertiary care medical center.

## Background

CMR is the test of choice for evaluating cardiac masses because it provides excellent tissue characterization of the mass in virtually any plane of the body. Biopsy proven cardiac masses provide information on the usefulness and accuracy of CMR in correctly diagnosing cardiac masses.

## Methods

Over a 7 year period, 161 patients were referred for CMR for evaluation of a known or suspected cardiac mass. A retrospective chart review of these 161 patients revealed that 42 patients (26%) had subsequent biopsy-proven diagnosis of a cardiac mass. CMR characteristics of biopsy proven cardiac masses, including contrast enhancement on first pass and delayed images, signal intensity on T1- and T2-weighted images, size, location, mobility and final CMR diagnosis were reviewed. CMR diagnostic performance was correlated with histopathologic diagnosis.

## Results

Pathologic diagnosis of tumor was established in 31 cases (74%), of which 19 (61%) were considered malignant. Diagnostic performance measures of CMR for distinguishing between tumor and non-tumor masses were as follows: sensitivity 94%, specificity 91%, PPV 97%, NPV 83%. Diagnostic performance measures of CMR for distinguishing between benign (tumor or non-tumor) and malignant masses were as follows: sensitivity 89%, specificity 100%, PPV 100%, NPV 92%. There were 2 malignant masses that were misclassified as benign on CMR, including 1 poorly differentiated sarcoma that was misdiagnosed as a vegetation, and 1 liposarcoma that was misdiagnosed as a myxoma. CMR provided the correct histopathologic diagnosis in 30/42 (71%) biopsy proven cardiac masses. In an additional 3 cases, CMR did not provide a specific diagnosis, but suggested a malignant neoplasm that was confirmed on pathology. In univariate analysis of CMR characteristics, the presence of increased T2 signal (r=0.46, p<0.005) and any contrast enhancement (r=0.78, p<0.001) was predictive of neoplasm, and lack of mobility (r=-0.62, p<0.001) and any contrast enhancement (r=0.46, p<0.01) was predictive of malignancy on pathology.

## Conclusions

Comprehensive cardiac MRI using cine, fast spin echo T1/T2 weighted, first-pass perfusion, and delayed enhancement imaging provides useful, diagnostic information in the evaluation of cardiac masses.

## Funding

NYU School of Medicine.

